# NK cells are never alone: crosstalk and communication in tumour microenvironments

**DOI:** 10.1186/s12943-023-01737-7

**Published:** 2023-02-16

**Authors:** Yongqiang Zhou, Lu Cheng, Lu Liu, Xun Li

**Affiliations:** 1 The First School of Clinical Medicine, Lanzhou University, 222 Tianshui South Road, Lanzhou, 730000 China; 2 Department of General Surgery, The First Hospital of Lanzhou University, Lanzhou, China; 3 Key Laboratory of Biotherapy and Regenerative Medicine of Gansu Province, Lanzhou, China; 4 Department of Pediatrics, The First Hospital of Lanzhou University, Lanzhou, China

**Keywords:** NK cells, Crosstalk, Communication, Cancer, TME

## Abstract

Immune escape is a hallmark of cancer. The dynamic and heterogeneous tumour microenvironment (TME) causes insufficient infiltration and poor efficacy of natural killer (NK) cell-based immunotherapy, which becomes a key factor triggering tumour progression. Understanding the crosstalk between NK cells and the TME provides new insights for optimising NK cell-based immunotherapy. Here, we present new advances in direct or indirect crosstalk between NK cells and 9 specialised TMEs, including immune, metabolic, innervated niche, mechanical, and microbial microenvironments, summarise TME-mediated mechanisms of NK cell function inhibition, and highlight potential targeted therapies for NK-TME crosstalk. Importantly, we discuss novel strategies to overcome the inhibitory TME and provide an attractive outlook for the future.

## Introduction

**Fig. 1:**
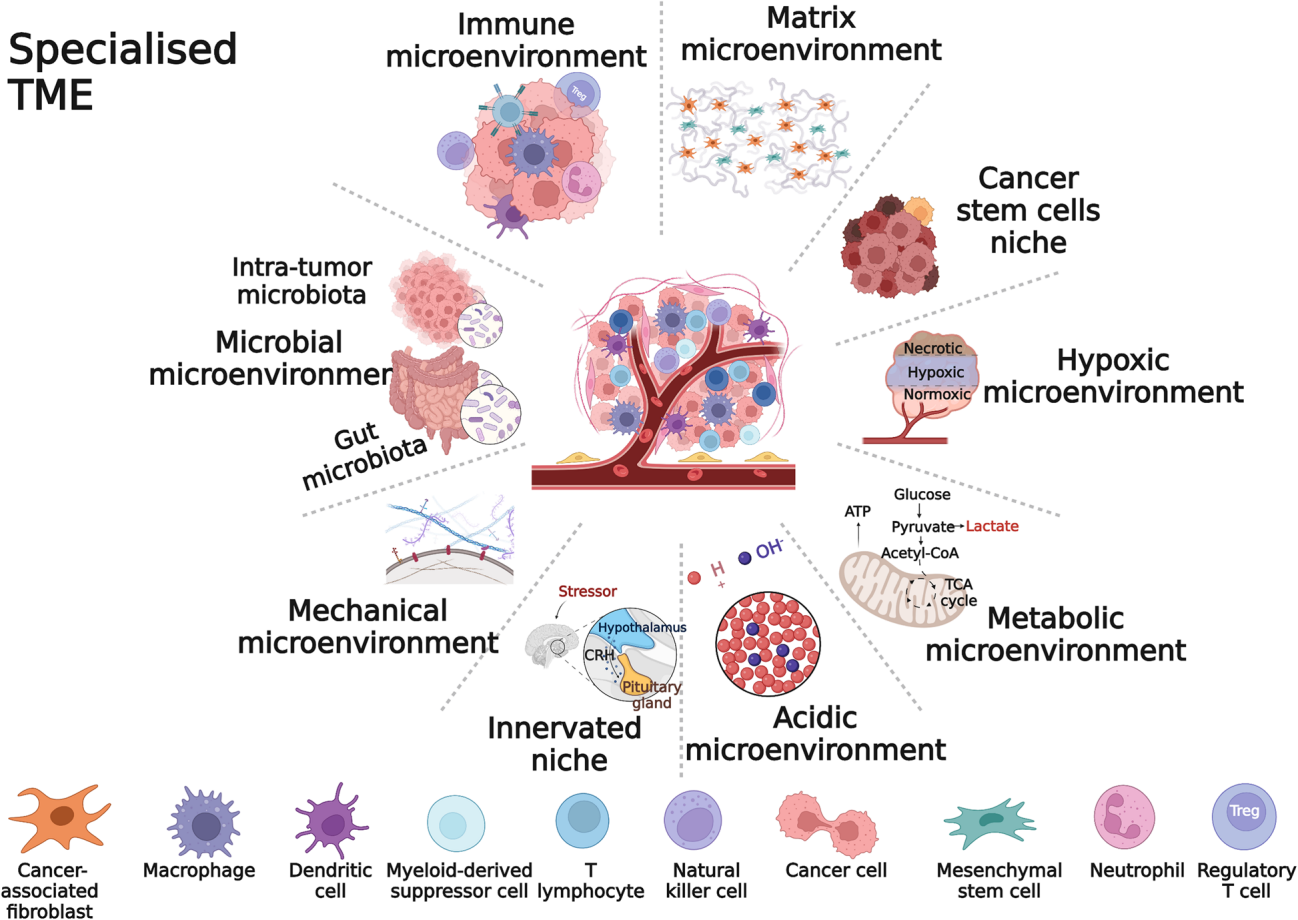
Specialised microenvironments. To characterise the NK cells crosstalk with the TME in more detail, the TME was subdivided into 9 specialised microenvironments. Different specialised microenvironment functions may overlap. The stromal microenvironment represents key stromal cells (tumor-associated fibroblasts and mesenchymal stem cells). *Adapted from “Forensic Analysis”, by BioRender.com (2022). Retrieved from *https://app.biorender.com/biorender-templates

Cancer is a dynamic and complex disease, and the development, progression and treatment of cancer require the involvement of the entire organism. Tumour formation is the result of the interaction of cancer cells with infiltrating immune cells, stromal cells, blood vessels, the extracellular matrix (ECM), secretory products (cytokines, chemokines, metabolites) and specific environmental conditions (e.g. hypoxia) [1]. All of these participants form a dynamic and heterogeneous biological network called the tumour microenvironment (TME) [2]. The TME assists cancer cells in evading host immunity, leading to tumorigenesis, progression, and metastasis. Targeting the TME represents a promising tumour therapy strategy [3]. In recent years, TME classification based on the functional TME specialisations has been gradually perfected and suggests the potential for combination therapy [4, 5]. Recently, the interaction of cancer-associated fibroblasts (CAFs), mesenchymal stem cells (MSCs), and cancer stem cells (CSCs) with immune cells has received increasing attention, so we further subdivided the TME into nine specialised TMEs to better characterise the state of immune cells in the TME. The specialised TMEs interact with each other and form a complete TME system (Fig. 1).

Cancer is a systemic disease, and a diverse portfolio of therapies, including surgery, chemotherapy, radiotherapy, immunotherapy, targeted therapy, cell therapy, and gene therapy, have become the primary treatments for cancer today. Cell immunotherapy-based cancer treatment has become a popular topic in drug research. Natural killer (NK) cells are a kind of natural cytotoxic lymphocyte, and due to their widespread antitumour effects, broad accessibility, and relative therapeutic safety, they are promising agents for cellular immunotherapy [6].

Preliminary data accumulated from preclinical studies and several clinical studies confirm that NK cell therapy shows therapeutic efficacy [7]. However, NK cell therapy still faces a number of challenges, such as insufficient NK cell infiltration, NK cell dysfunction, and metabolic dysregulation [8]. These challenges are a result of NK cell sensitivity to multiple immunosuppressive mechanisms that are active in the TME. The mechanism by which NK cells crosstalk with the TME and how to overcome it is a primary topic of discussion for many researchers.

Here, we outline the biological functions of NK cells and highlight their unique ability to kill cancer cells. Then, we review the function of each specific TME and describe the detailed NK cell crosstalk to show the potential therapeutic value of targeting NK-TME crosstalk. Finally, we describe current strategies to overcome the inhibitory effects of the TME and suggest potential solutions for addressing several current issues.

## Killing and recognition of NK cells

NK cells have been identified as CD3^−^CD56^+^ cells and can be further subdivided into two major subgroups based on CD56 expression: CD56^bright^ and CD56^dim^ NK cells [9]. CD56^bright^ NK cells are relatively immature NK cells that account for approximately 5–10% of peripheral blood NK cells and are generally thought to play immunomodulatory roles by producing cytokines. CD56^dim^ NK cells are fully mature NK cells that account for approximately 90–95% of peripheral blood NK cells, and they kill target cells directly through antibody-dependent cell-mediated cytotoxicity (ADCC) [10, 11]. CD56^bright^ NK cells are mostly found in secondary lymphoid organs such as lymph nodes and tonsils, while CD56^dim^ NK cells are found in peripheral blood, bone marrow, and peripheral organs like the spleen and lungs [12]. Although defined as a different subtype, CD56^bright^ NK cells are also considered a precursor of CD56^dim^ NK cells. After exposure to interleukin (IL)-2 and/or IL-15, CD56^bright^ NK cells eventually differentiate into CD56^dim^ NK cells [13].

NK cells directly kill cells identified as cancerous, infected, or stressed by releasing cytolytic particles containing perforin and granzyme B. In addition, NK cells induce apoptosis by expressing death receptors such as Fas ligand (FasL) and TNF-related apoptosis-inducing ligand (TRAIL) [14].

The process by which NK cells recognise "self" and "nonself" does not require somatic gene rearrangement to produce clones that recognise different antigens but does require precise control of NK cell function through coordinated regulation of receptor activation and inhibition [15]. Specifically, when NK cell surface inhibitory receptors, mainly killer immunoglobulin-like receptors (KIRs), leukocyte Ig-like receptors (LIRs/ILTs), and the CD94/NKG2 family of C-type lectin receptors, recognise major histocompatibility complex class I molecules (MHC-I) through "the education" process [16], NK cells become inactivated and build self-tolerance [17, 18]. And when NK cells recognise more activating receptors, primarily natural cytotoxic receptors (NCR), DNAX accessory molecule 1 (DNAM-1), and natural killer group 2 member D (NKG2D) through the "missing self" or "induced self" mechanism, they trigger target cell lysis [19].

In summary, unlike T cells, NK cells do not require tumour-specific recognition and are not limited by MHC inhibition. The widespread antitumour effects of NK cells, which directly detect and destroy cancer cells, make them promising candidates for tumour immunotherapy.

## Intercellular communication between NK cells and cancer cells

### Gap junctions

**Fig. 2:**
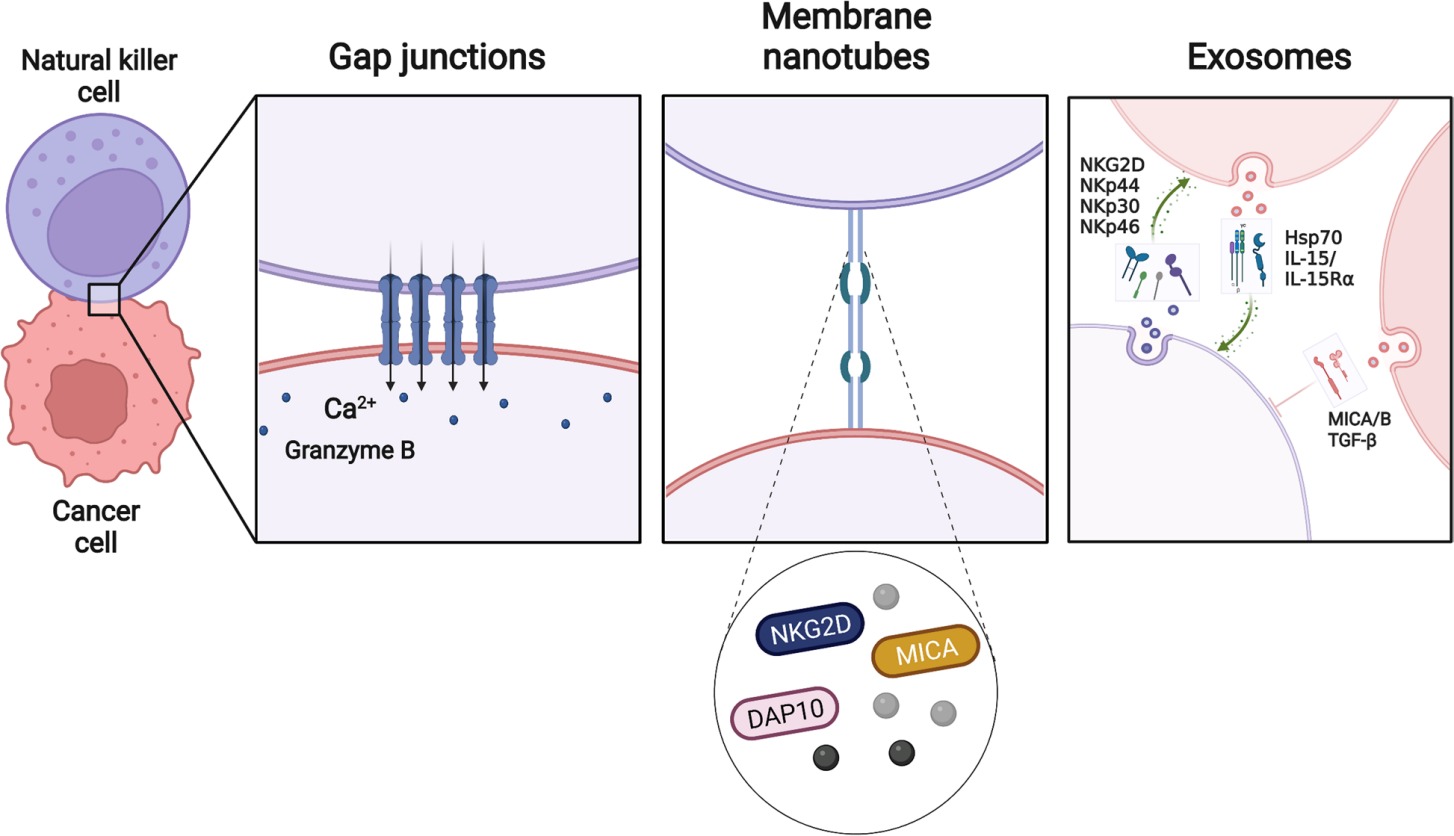
Intercellular communication between NK cells and cancer cells. Gap junctions allow NK cells to communicate directly with cancer cells, allow the influx of Ca2^+^ into cancer cells, and promote granzyme B activity and cancer cells apoptosis. Membrane nanotubes allow NK cells to communicate remotely with cancer cells, where proteins and cytokines accumulate to mediate the lysis of distant cancer cells. Exosomes mediate two-way communication between cancer cells and NK cells and are important mediators of intercellular communication. DAP10, DNAX-associated protein 10. NKG2D, Natural killer group 2 member D. MICA/B, MHC class I chain-related protein A/B. Hsp70, Heat-shock protein 70. NKp44/30/46: Natural cytotoxicity receptor 44/30/46*. Created with BioRender.com*

Gap junctions (GJs) are hydrophilic channels that connect adjacent cell membranes, allowing them to communicate directly. Connexins (Cx) are an integral transmembrane protein family in GJs that allows the bidirectional transfer of glutathione, glucose, glutamate, adenosine triphosphate (ATP), cyclic adenosine monophosphate (cAMP), inositol triphosphate (IP3), ions, micropeptides (including antigens), and microRNAs through these communication channels. Connexin 43 (Cx43) is the major GJ protein in the immune system [20]. A bidirectional GJ coupling exists between NK cells and cancer cells. Increased Cx43-GJ cell-to-cell communication was observed in cytotoxic immune synapses (IS) formed between NK cells and cancer cells, allowing Ca2^+^ to flow into the cancer cells and promoting granzyme B activity and cancer cell apoptosis [21] (Fig. 2). The classical granzyme A and granzyme B apoptotic pathways are Ca2^+^-dependent processes in which perforin in the cell allows Ca2^+^ to pass through small holes in the cancer cell membrane, and Ca2^+^ flows into the membrane, triggering the damaged membrane response and initiating the cancer cell apoptosis process [22]. Thus, Cx43-GJ intercellular communication is a key component that leads to the programmed death of cancer cells.

Intercellular communication via Cx43-GJ is essential for NK cell-mediated tumour immunity and NK cell function. Disruption of intercellular communication or low expression of Cx43-GJs has been shown to strongly inhibit granzyme B activity and Ca2^+^ influx, as well as NK cell-mediated cancer cell death [21, 23].

### Membrane nanotubes: long-range attack weapons

Membrane nanotubes are ubiquitous intercellular signalling structures that transport multiple signalling molecules over long distances to aid intercellular communication. Studies have shown that between NK cells and target cells, membrane nanotubes can form where proteins accumulate, allowing distant interactions between NK cells and target cells and leading to the lysis of distant target cells [24] (Fig. 2). Other studies have shown that maternal decidual NK (dNK) cells can control bacterial infection by forming nanotubes that attach to trophoblast cells, deliver the antimicrobial peptide granulysin, and kill bacteria that infect the cells [25]. The frequency of nanotube formation depends on the number of receptor-ligand interactions. However, only specific ligand-receptor pairs promote nanotube formation [26]. Although the application of membrane nanotubes to tumour immunotherapy remains to be determined, the presence of nanotubes may enable NK cells to maintain interaction with a specific moving target or patrol multiple targets at once [24]. Membrane nanotubes help NK cells better mediate cytotoxicity and represent a novel immunomodulatory approach.

### Exosomes: Mediators between NK cell and cancer cell intercellular communication

Exosomes are cell-secreted nanosized vesicles that carry proteins, lipids, and nucleic acids and act as mediators in the autocrine, paracrine, or endocrine signalling of intercellular substances [27].

As Batista et al. have observed, exosomes are an important intercellular communication system between NK cells and cancer cells [28]. Tumour-derived exosomes (TDEs) affect NK cell biology by fusing with the NK cell membrane and internalising their contents and play a decisive role in immune escape and cancer progression [29]. TDEs have a dual role in regulating NK cell activity. On one hand, TDEs enhance NK-mediated cell death through Hep protein or IL-15/IL-15Rα expression [30, 31]. On the other hand, TDEs can inhibit NK cell activity through the expression of TDE inhibitory biomolecules [29] (Fig. 2).

NK cell-derived exosomes (NK-Exos) possess some of the characteristics of NK cells. In 2017, a simple and large-scale method for NK cell isolation was proposed, and NK-Exos were shown to contain the cytotoxic proteins perforin, granulysin, and granzymes A and B [32]. Moreover, NK-Exos induce cancer cell apoptosis in a caspase-dependent manner via receptor-ligand interactions (e.g., Fas/Fas-L) [33]. They express the typical NK cell markers CD16, CD69, NKp44, and NKG2D. DNAM1 and activate caspase-dependent apoptosis to mediate the interaction between exosomes and cancer cells [34]. In addition, Furthermore, NK-Exos influence immune responses in cancer progression via the tumour suppressor miR-186 [35]. It has also been shown that hypoxia affects the biogenesis of NK-Exos and is able to increase their production and cytotoxicity [36].

Importantly, engineered NK-Exos have been developed to improve antitumour outcomes. For example, NK-Exos loaded with small interfering RNA from BCL-2 enhanced intrinsic apoptosis in breast cancer (BC) cells [37]. Combining NK-Exos with biomimetic core–shell nanoparticles could be an effective strategy for antitumour therapy [38].

Overall, the above insights highlight the communication between NK-Exos and cancer cells, although the underlying mechanisms remain to be explored.

## Crosstalk between NK cells and the TME

**Fig. 3:**
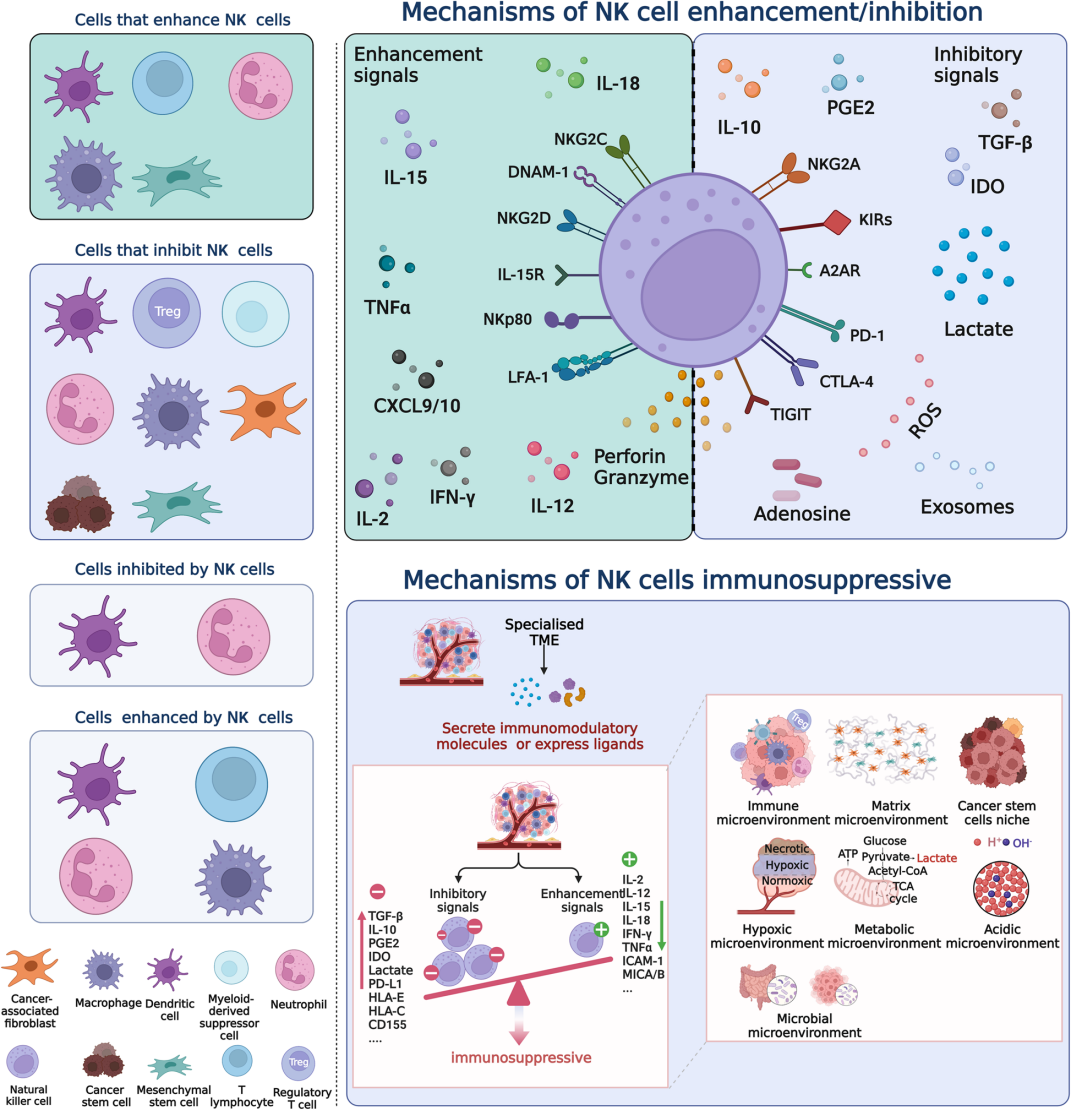
Crosstalk between NK cells and the TME. In the TME, complex crosstalk exists between NK cells and key cellular components in TME, and this interaction affects their respective cellular functions (left panel). In the intricate TME, exposure to enhancement signals promotes NK cell antitumor responses, and conversely, exposure to inhibitory signals puts NK cells in an immunosuppressive state. NK cells are in a dynamic microenvironment, and the functional transition that occurs when NK cells crosstalk with a specialised microenvironment depends on the balance of enhancement signals and inhibitory signals. Specialised microenvironments involve the immune microenvironment, the matrix microenvironment, the cancer stem cell niche, the hypoxic microenvironment, the metabolic microenvironment, the acidic microenvironment, and the microbial microenvironment (right panel). TNFα, Tumor necrosis factor α. IFN-γ, Interferon-γ. IL-15/2/12/18/10, Interleukin-15/2/12/18/10. PGE2, Prostaglandin E2. IDO, Indoleamine 2,3-dioxygenase. TGF-β, Transforming growth factor-β. NKG2A/C/D, Natural killer group 2 member A/C/D. ROS, Reactive oxygen species. TIGIT: T-cell immunoglobulins and ITIM domain. CTLA-4, Cytotoxic T-lymphocyte antigen 4. PD-1, Programmed death-1. A2AR, A2A adenosine receptor. KIRs, Killer immunoglobulin-like receptors. NKp80: Natural cytotoxicity receptor 80. IL-15R, Interleukin 15 receptor. DNAM-1, DNAX accessory molecule 1. LFA-1, Lymphocyte function-associated antigen-1. ICAM-1, Intercellular adhesion molecule-1. MICA/B, MHC class I chain-related protein A/B. PD-L1, Programmed cell death ligand 1. HLA-C/E, Human leukocyte antigen C/E. CXCL9/10, Chemokine (C-X-C motif) ligand 9/10. *Adapted from “The Key Role of Neuroinflammation in Neurodegenerative Diseases”, by BioRender.com (2022). Retrieved from *https://app.biorender.com/biorender-templates

Growing evidence suggests that NK cells exhibit complex crosstalk with various specialised TMEs. NK cells and specialised TMEs sometimes cooperate with each other, but more often they fight against each other, thereby altering the antitumour response. The balance of a variety of activating and inhibitory signals determines the outcome of the interaction (Fig. 3), and overcoming the immunosuppressive TME has become the focus of current cancer treatment strategies.

### Immune microenvironment: crosstalk between NK cells and other immune cells

#### Crosstalk between NK cells and T cells

In complex immune microenvironments, NK cells are able to enhance T-cell effector functions and work together to effectively regulate antitumour immunity. In the immune cycle of cancer-NK cells, NK cells are responsible for recognising and killing sensitive cells that are under stress or undergoing an early malignant transformation. However, cancer cells' evolution leads to the redistribution of NK cell ligands, which alters their sensitivity to NK cells. This evolution enables NK cells to drive tumour inflammation by producing chemokines and conventional type 1 dendritic cells (cDC1) to recruit and present tumour neoantigens and present them to CD8^+^ T cells in lymph nodes. Finally, neoantigen-specific CD8^+^ T cells induce antitumour immunity and tumour regression [39]. In MHC-induced immune evasion, cancer cells evade the recognition of cytotoxic CD8^+^ T cells by downregulating MHC-I expression. However, downregulation of MHC-I leads NK cells to activate a "missing self" mechanism to lyse cancer cells. In this case, NK cells and T cells complement each other in MHC-induced immune evasion, and their joint "fail-safe" mechanism can be exploited for therapeutic purposes [40].

Accumulating evidence suggests that NK cells either enhance or impair T cell responses in a direct or indirect manner. In terms of direct mechanisms, IL-12 or IL-2-conditioned NK cells, provide naive T cells with interferon-γ(IFN-γ), which is required for the induction of T helper cell type 1 (Th1) polarization [41]. Activated NK cells expressing OX40 ligand and B7 induce the proliferation of autologous T cells [42]. T cells' released cytokine IL-2 has been shown to play a role in NK cell activation [43, 44]. Further, mutual cooperation between DNAM-1 and NKG2D negatively affects T-cell responses [45]. Recent studies have shown that indirect mechanisms are involved in NK cell-T cell crosstalk, where dendritic cells (DCs) are the bridge that mediates this interaction. Activated NK cells after exposure to MHC class I^low^ cancer cells initiate DC production of IL-12 and induce enhanced CD8^+^ T cell-mediated tumour control [46]. This finding is supported by the fact that activated NK cell-mediated IFN-γ secretion stimulates IL-12 production by DCs and, ultimately, initiates CD8^+^ T antitumour responses in A20 B-cell lymphoma cells [47]. In addition, it has been shown that the interaction of NK cells with myeloid-derived suppressor cells (MDSCs) is involved in indirect mechanisms of T cell-NK cell crosstalk. Activated NK cells after cryo-thermal therapy reduced MDSCs accumulation via NKG2D-NKG2D ligands axis and reprogrammed MDSCs to a mature phenotype via IFN-γ, promoting CD4 Th1-dominant antitumour immunity in the B16F10 melanoma model [48]. Notably, evidence of bidirectional crosstalk between NK cells and T cells has emerged. The roles of cytokines released from T cells, such as IL-2 and IL-15, in NK activation are well established [49].

T-cell-NK cell crosstalk has been proposed to be associated with favourable disease outcomes. In colorectal cancer (CRC), infiltration of both NK cells and CD8^+^ T cells is associated with prolonged patient survival [50]. Tumour-specific T cells have been shown to support NK cell elimination of mouse mastocytom [51]. Cetuximab-activated NK cells trigger DC maturation and tumour antigen-specific T cell immune responses in patients with head and neck cancer (HNC) [52].

Sufficient mobilisation of T-NK cell synergy offers broad prospects for ameliorating MHC-I deficiency and poor immune cell infiltration prevalent in immunosuppressive TME and immunotherapy. For example, the activation of NK cells observed in the mouse model depended on the reactivation of CD8^+^ T cells at the tumour site, whereas the collaboration of T cells and NK cells directly led to the elimination of cancer cells [51]. Cancer vaccines targeting MHC class I chain-related protein A/B (MICA/MICB) stress molecules that activate synergistic attacks by T cells and NK cells have demonstrated efficacy and safety in mice and rhesus macaques [53]. A STING agonist (activating antitumour T-cell response) in combination with H9 superkine (IL-2 variant expressed by NK cells with a high affinity for the IL-2R β/γ complex) showed strong synergistic effects. Mobilization of T and NK cells was found to be effective in both refractory MHC I-deficient and MHC I^+^ tumours in mice [54].

#### Crosstalk between regulatory T cells (Tregs) and NK cells

Although NK cells are active in antitumour immunity, they are sensitive to suppressive components of TME, including Tregs [55]. Inhibition of NK cell function by Tregs was observed as early as 1999 [56]. Similarly, in 2004, Trzonkowski et al. observed that human NK cell activity was inhibited in the presence of Tregs [57]. In 2005, Ghiringhelli et al. first reported the mechanism of Treg-NK interactions. They observed that Tregs inhibited NK cell responses directly via membrane-bound transforming growth factor (TGF)-β and that Tregs deletion restored NK cells' ability to mediate human cancer cells lysis [58]. Since then, a growing number of studies have found that TGF-β plays important roles in Tregs' inhibition of NK cells function. In non-small-cell lung cancer (NSCLC) patients, Tregs effectively inhibit the antitumour activity of autologous NK cells, and TGF-β is thought to be the main mechanism of NK cells inhibition [59]. Indeed, Tregs interfere with NK cells via a variety of mechanisms, including TGF-β-induced downregulation of NKG2D expression in an in vitro co-culture model [60]. Notably, in some cases, NK cells are resistant to Tregs. Specifically, NK cells contain two phenotypes: canonical and adoptive; adaptive NK cells are inherently resistant to Treg inhibition [61].

In addition, enriched ATP in the TME can be degraded to cAMP by CD39, and subsequently, CD73 expressed on the surface of Tregs can convert cAMP to adenosine, which binds to the A2A adenosine receptor (A2AR) on NK cells to negatively regulate NK cell maturation and antitumour response [55]. It has also been shown that the negative regulation of NK cells by Tregs largely depends on cytokine levels in the environment. Tregs inhibit NK cells through their high consumption of IL-2, and under conditions of substantial IL-2 stimulation, Treg inhibition can be reversed and NK cell responses can be activated during an acute retroviral infection [62]. Similarly, it has been observed that stimulation of IL-12 and IL-18 eliminates Treg inhibition and enhances NK cell cytotoxicity [63].

Targeting Tregs in the TME is certainly a promising therapeutic option. Targeting cytotoxic T-lymphocyte antigen 4 (CTLA-4)^+^ Tregs with ipilimumab restored NK cell-mediated ADCC and enhanced antitumour immunity in HNC [64]. Moreover, the combination of the anti-programmed death-1 (PD-1) antibody Nivolumab with the anti-C–C chemokine receptor type 4 (CCR4) antibody Mogamulizumab (a Treg-depleting antibody) showed promising antitumour activity and supported potential therapeutic options [65]. The use of signal transducer and activator of transcription 3 (STAT3) antisense oligonucleotides in murine orthotopic pancreatic adenocarcinoma models further validated the therapeutic potential of targeting Tregs. The results of Piper et al. suggest that targeted inhibition of STAT3 on Tregs results in enhanced NK-mediated immunosurveillance of metastasis [66].

#### Crosstalk between DCs and NK cells: bidirectional interactions

DCs are a relatively small group in the TME immune cell population but play essential roles in tumour immunity due to their unique antigen-presenting function and immunomodulatory function through intercellular contact and cytokine production [67]. In 1999, Fernandez et al. first demonstrated cell-to-cell contact between DCs and NK cells [68]. Recent studies have clearly illustrated the bidirectional crosstalk between DCs and NK cells. The bidirectional crosstalk between NK cells and DCs can activate NK cells and enhance antitumour activity [69, 70]. Moreover, NK cells also affect the recruitment [71, 72] and maturation [73] of DCs. FMS-related tyrosine kinase 3 ligand (FLT3L) is essential for the in situ development and proliferation of conventional DCs (cDCs) [67, 74] and is mainly produced by NK cells in the TME [75]. Cazzetta et al. reported that DCs release cytokines and chemokines such as IL-12, IL-15, IL-18, IFN-γ, and chemokine (C-X-C motif) ligand 9/10 (CXCL9/10) to promote NK cell activation and recruitment to sites of inflammation. Furthermore, activated NK cells produce IFN-γ, tumour necrosis factor α (TNF-α), and XCL1 to promote DC maturation and recruitment [76]. Similarly, the data from Bottcher et al. also showed that NK cell-derived CCL5 and XCL1 promote cDC1 migration to tumours and induce cDC1 accumulation in the TME to improve tumour immune control [71]. This study highlights the importance of DC-NK cell crosstalk in the antitumour immune response. This bidirectional crosstalk between DCs and NK cells forms a positive feedback loop, and this bilateral activation and recruitment constitutes a powerful immune regulatory network in the TME. Interestingly, NK cells can also serve as mediators of DC-T-cell interactions [77]. NK cells appear to regulate the TME in a dynamic fashion [78].

Notably, the production of prostaglandin E2 (PGE2) in cancer cells can reduce the accumulation of cDCs by impairing NK cell viability and chemokine production, causing immune evasion [71]. Similarly, the production of IL-6 and IL-10 in cancer cells and immune cells in the TME also causes DC dysfunction [79]. Upregulation of CTLA-4 expression on NK cells has a negative impact on DC maturation in human NSCLC [80]. Studies have also shown that DCs may impair NK cell function [81].

In conclusion, the crosstalk between NK cells and DCs is critical for regulating antitumour immunity and may be a promising target for effective antitumour therapy [82]. For example, in an orthotopic liver tumour model, CD47 blockade enhanced antitumour efficacy by triggering the DC- NK cell axis [83]. Moreover, CD47 blockade enhanced CD103 DC phagocytosis and induced CXCL9 and IL-12 secretion through the STING pathway to promote NK cell recruitment and activation [83]. The above evidence highlights that the CD103-DC-NK cell axis can be an independent pathway in antitumour process. Similarly, cooperation between DCs and NK cells has been shown to promote antitumour immune responses in experimental lymphomas [84].

#### Crosstalk between neutrophils and NK cells.

As early as 1863, Virchow proposed that inflammation is a risk factor for tumours formation [85]. In 1986, Harold F. Dvorak wrote, based on Virchow's hypothesis, "tumors are incurable wounds" [86]. When the body suffers from inflammation, neutrophils are the first to respond, and a large number of neutrophils infiltrate in tumours during chronic inflammation [87]. NK cells and neutrophils are colocalized in the TME, suggesting that they participate in mutual crosstalk [88].

Neutrophils are traditionally considered to have both promotive [89] and suppressive [90] roles in primary tumours. However, most studies on neutrophils have been conducted in specific environments. Li et al. discussed the tumour regulatory role of neutrophils under the same conditions [91]. Interestingly, neutrophils exhibit inhibition of tumour metastasis when NK cells are absent and activation of tumour metastasis in the presence of NK cells in mouse models of BC, suggesting that the abundance of host NK cells governs the dual role of neutrophils. Importantly, reactive oxygen species (ROS) also mediate this dual regulation of neutrophils [91]. Similarly, the study of Ogura et al. supports that NK cells control neutrophils' tumour regulation. However, neutrophils exhibited pro-tumour effects in the absence of NK cells [92]. The dual identity of neutrophils is attributed to their heterogeneity and functional plasticity in different environments; they can dynamically change to adapt to different environments.

Defining neutrophils that exhibit pro-tumour or anti-tumour effects is difficult, and characterising neutrophil-NK cell crosstalk is a daunting task. Accumulated evidence has shown that soluble mediators, cell-to-cell interactions, and extracellular vesicles (EVs) mediate neutrophil-NK cell crosstalk (reviewed in Scapini et al. [93]). Specifically, neutrophils have been shown to attenuate NK cell infiltration through downregulation of CCR1 in a mouse model of CRC while inhibiting the responsiveness of NK cell activating receptors NKp46 and NKG2D [94]. The underlying cause of impaired NK cell immunity is PD-L1/PD-1 interaction, which results in lower IFN-γ levels [94]. Neutrophils mediate NK cells' responses by releasing different molecules, including cathepsin G [95] and ROS [96], to regulate NKp46 expression. Neutrophil-derived EVs enhance the production of anti-inflammatory cytokines in NK cells [97]. Further, neutrophil-NK cell crosstalk serves as a key step in promoting the invasion-metastasis cascade response. In a mouse model injected with 4T1 breast cancer cells, neutrophils inhibited NK cell activity, thereby attenuating NK cell-mediated clearance of intraluminal cancer cells [98].

The role of IFN-γ as a key mediator of neutrophil-NK cell crosstalk is gaining prominence. IFN-γ is responsible for the increased neutrophil survival observed when co-cultured with NK cells in the presence of IL-2 and IL-15 [99]. In a transplantable model of sarcoma, it was reported that NK cells regulate neutrophil function through an IFN-γ-mediated pathway. Upon NK cell depletion, neutrophils produce elevated levels of VEGF-A and acquire a tumour-promoting phenotype [92]. In addition, in the context of inflammation, neutrophil-derived arginase I inhibits the release of IFN-γ from NK cells [100]. Importantly, IL-12 is required for IFN-γ expression in both human and murine NK cells, implying that neutrophils control NK cells via IL-12 production [101]. Neutrophils affect NK cell recruitment and/or accumulation in lymph nodes and contribute to IFN-γ production in a DC-vaccinated mouse model [102].

NETosis is a specific form of neutrophil death in which the nuclear membrane ruptures and the extruded nuclear DNA and cytoplasmic granule proteins form neutrophil extracellular traps (NETs) to capture and kill pathogens [103]. NETs have pro-tumour functions through a variety of mechanisms [104, 105]. Teijeira et al. investigated the relationship between NETs and the tumour immune microenvironment. NETs were found to cover tumour cells and form a physical barrier between tumour cells and immune cells, protecting tumour cells from CD8^+^ T cells and NK cells. Interestingly, blocking NETosis inhibits tumour metastasis only in the presence of NK cells [106]. These data suggest that NETs are capable of blocking the tumoricidal effects of NK cells and provide evidence that NETs interfere with the contact of NK cells with cancer cells.

#### Crosstalk between macrophages and NK cells: adapt and react on demand

Macrophages are the most abundant subset of immune cells in many tumour types and are present in all stages of tumour progression, playing an essential role in tumour immune regulation. Macrophages are directly or indirectly involved in several key features of malignant tumours, including angiogenesis, metastasis, immunosuppression, and treatment resistance [107].

Macrophages exhibit great heterogeneity and a highly plastic phenotype in the TME. Numerous studies support the hypothesis that macrophages initiate, promote or inhibit tumour development by secreting cytokines and establishing intercellular contact [108]. However, support for the dual regulatory role of macrophages has mostly been studied in a binary M1/M2 context, and it has been argued that macrophages do not refer to a single cell population but to a collection of multiple cell types with a wide range of functional roles. Therefore, defining the functional state of macrophages from a single perspective alone is not sufficient [109]. For example, tumour-associated macrophages (TAMs) are more likely to have M2 phenotypes, but TAMs can polarise into both M1 and M2 phenotypes [110]. To this extent, it is necessary to understand immune regulation in the TME from the perspective of crosstalk between macrophages and other cells.

As reviewed by Zhou et al., macrophages and NK cells crosstalk through different mechanisms [111]. Soluble mediators play a central role in macrophages and NK cells crosstalk. In the TME, macrophages release activating cytokines (e.g., IL-12, IL-15, IL-18 and TNF-α), which promote antitumour cytotoxicity of NK cells [112]. TGF-β is a major inhibitory cytokine released by macrophages that inhibits NK cell effector functions by inhibiting NK cell activating receptors (e.g., NKG2D and NKp30) [113], regulating chemokine receptor expression (e.g., CX3CR1) [114], promoting inhibitory receptor expression (e.g., ILT-2) [115], and decreasing NK cell-mediated cytokine release (e.g., IFN-γ and TNF-α) [116]. As a pleiotropic cytokine, IL-10 secreted by macrophages exhibits a dual role in regulating NK cell function. IL-10 has been shown to enhance the lytic activity of NK cell [117]. IL-10 increases the cytolytic activity of human NK cells in the presence of IL-15 [118]. Combination with IL-18 has also been shown to enhance NK cell proliferation and cytotoxic activity [119]. Furthermore, the NK cell-derived cytokine IFN-γ induced M1 macrophages [120]. In prostate cancer, peripheral blood tumour-associated circulating NK (pTA-NK) cells induce M2-like features in vitro [121]. Furthermore, receptor-ligand binding establishes bidirectional crosstalk between NK cells and macrophages [111].

There is extremely complex crosstalk between macrophages and NK cells in the TME. Investigating the vast landscape of macrophage-NK crosstalk networks can provide new perspectives on immunotherapeutic strategies. Several promising new therapies have already emerged. For example, to dismantle TAM-mediated immunosuppression, Eisinger et al. used antibodies specifically targeting the TAM scavenger receptor MARCO to activate the NK cell-mediated cell death of human melanoma cells [122].

### Matrix microenvironment: focusing on CAFs and MSCs

#### Crosstalk between CAFs and NK cells

CAFs are one of the most important matrix components in the TME and play a wide range of roles in ECM remodelling, cancer cell proliferation, drug resistance, invasion, and metastasis [123]. CAFs can be derived from a variety of cell types and cells at different stages of differentiation. Cell subpopulations with different phenotypes and functions within the same tumour type also contribute to the biological heterogeneity of CAFs. The biological heterogeneity of CAFs determines the complex interaction between CAFs and cancer cells. Generally, most CAF subgroups act as cancer-promoting CAFs (pCAFs) that promote tumour progression, while cancer-restraining CAFs (rCAFs) inhibit tumour progression [124]. It has also been reported that the cellular state of fibroblasts determines the CAF phenotype [125].

CAFs can inhibit NK cell-mediated cancer cell death in multiple ways, including the expression of soluble mediators, modulation of NK cell activation receptors, and synergy with other immune cells (reviewed in Mao et al. [124]). For example, PGE2 and indoleamine 2,3-dioxygenase (IDO), both derived from hepatocellular carcinoma-associated fibroblasts, create an unresponsive state in tumours by "educating" NK cells to have an inactive phenotype [126]. CAFs isolated from endometrial cancer tissue inhibit NK cell-mediated cell death through downregulation of the ligand poliovirus receptor (PVR/CD155), which activates the NK cell receptor DNAM-1, thereby aiding cancer progression [127]. It has also been shown that human CRC-derived CAFs inhibit NK cell function by promoting the recruitment of M2 macrophages in tumour tissues and by synergizing with other immune cells [128]. These studies reveal complex mechanisms of crosstalk between CAFs and NK cells in the TME.

TGF-β is becoming increasingly important cytokine mediating cross-talk between CAFs and NK cells [40]. TGF-β is primarily produced by CAFs, and it is widely assumed that TGF-β can inhibit NK cells responses via a variety of mechanisms, include interfering with cytokine secretion (e.g., IFN-γ) [129], downregulating NK cell activating receptor expression (e.g., NKG2D, NKp30, and NKp44), and limiting NK cell metabolic activity [130]. TGF-β signaling inhibition has been shown to improve NK cell-mediated antitumour effects in a variety of preclinical tumour models, including lung cancer [131], triple-negative breast cancer [132], and a mouse liver metastases model of CRC [133].

Targeting CAFs may be an effective strategy to alleviate impaired NK cell function in the TME. In pancreatic ductal adenocarcinomas, blockade of CAF-related signalling has been shown to support NK cell activation and inhibit tumour metastasis [134]. Similarly, in CRC, targeting CAFs can counteract its inhibitory effect on NK cell function [135].

#### Crosstalk between MSCs and NK cells: Dual immunomodulatory effects

MSCs are pluripotent stem cells capable of producing multiple mesenchymal lineages, making them key regulators of tumour fate due to their strong tumour adaptation and differentiation potential [136]. Recent data highlight the immunomodulatory function of MSCs, which can interact with a wide range of immune cells, including T cells, B cells, dendritic cells, and NK cells [137].

A complex network of interactions exists between MSCs and NK cells. Numerous studies support the inhibition of NK cells by MSCs. They suppress NK cell function by producing and releasing soluble factors such as TGF-β, PGE2, IDO, human leukocyte antigen (HLA)-G, and exosomes [138, 139]. Moreover, direct cell-to-cell contact between MSCs and NK cells can inhibit NK cell function by downregulating NK cell surface activating receptors or reducing the release of perforin [140, 141]. In addition, the interference of MSCs with the intracellular signal transduction of NK cells and the interaction of MSCs with other immune cells (such as Tregs) are also important mechanisms by which MSCs inhibit the function of NK cells [142, 143].

However, some contradictory views cannot be ignored. MSCs increased the ability of IL-12/IL-18-stimulated NK cells to secrete IFN-γ in a dose-dependent manner, according to Thomas H et al. [144]. At lower doses, MSCs promoted NK cell formation and enhanced their antitumour activity against cancer cells, while at higher doses, MSCs inhibited NK cell formation and attenuated their tumour-killing effects [145]. A reasonable explanation for this dual immunomodulatory effect of MSCs on NK cells is that experimental conditions, including the MSC:NK cell ratio, cell concentration, quality of prestimulated NK cells, and coculture incubation time, affect the direction of interaction between MSCs and NK cells [138, 146]. It has also been suggested that MSCs are inherently heterogeneous and simultaneously release proinflammatory cytokines and immune cell suppressive factors [147].

MSCs inhibit NK cell function, but they are also targets for NK cell lysis. Previous data suggest that NK cells activated by different cytokines can effectively lyse MSCs, allowing the NK cells to penetrate the tumour stroma and maintain regulation of cancer cell death. However, quiescent NK cells do not have this potential [140]. Moreover, MSCs are easily cleaved by NK-92 cells (similar to activated primary NK cells) but are not readily cleaved by KHYG-1 cells (naive NK cells), indicating that MSCs have different crosstalk between the two NK cell lines [148]. Possible explanations are that multiple receptors (NKp30, NKG2D, and DNAM-1) are involved in NK cell-mediated MSC lysis and that the ligands of NK cell-activating receptors expressed by MSCs can trigger or inhibit the activation of NK cells, as well as the observed susceptibility of MSCs to lysis in different contexts [148, 149].

However, an inescapable question is how MSCs evade lysis by NK cells to maintain the regulation of NK cells. It has been observed that both autologous and allogeneic MSCs are susceptible to lysis by NK cells, suggesting that downregulation of HLA-I is not sufficient to protect MSCs from NK cell-mediated cell death [150]. Similarly, TLR3-induced HLA-I upregulation does not play a major role in the protection of MSCs [151]. The above results suggest that there must be other mechanisms for MSC protection. MICA and other NKG2D ligands have been shown to be involved in the protection of NK cell-mediated killing [151]. In addition, monocytes can act as a barrier between NK cells and MSCs, and monocytes can protect the proliferation and differentiation of MSCs by secreting key cytokines [152].

In conclusion, the crosstalk between MSCs and NK cells is complex. The interaction between MSCs and NK cells affects their respective functions, and given the important immunomodulatory functions of both cells, the impact of the interaction between MSCs and NK cells needs to be considered in the future development of cell-based therapies.

### Cancer stem cell (CSC) niche

Dormant stem cells hidden within tumours, called cancer stem cells (CSCs), are characterised by self-renewal and multidirectional differentiation and are often considered to be the main cause of tumour metastasis, recurrence, and treatment failure [153]. CSCs and the TME establish a wide range of interactions to form a special niche, known as the CSC niche [154]. The CSC niche is considered to be a component of tumour growth and metastasis. Understanding the complex interactions between immune cells and the CSC niche also helps to describe the mechanisms by which tumours evade immune surveillance [155].

NK cells have the ability to recognise and lyse CSCs, and allogeneic NK cells have been shown to preferentially target cancer cells with a CSC phenotype in several different cancer types (e.g.,melanoma, CRC, glioblastoma, and BC) [156]. The molecular mechanism underlying NK cell-mediated CSC recognition is that CSCs lack HLA I molecules and express various ligands that activate NK receptors (such as PVR, Nectin-2, NKp30, NKp44, ULBP1, ULBP2, MICA/B, Fas, and DR5), thereby triggering NK cytotoxicity [157–160]. Markers expressed by CSCs can also increase the CSC's sensitivity to NK cell lysis by stimulating NK cell activation [156].

However, the lysis of CSCs by NK cells is controversial. For example, no significant difference in lysis sensitivity between CSCs and non-CSCs was observed in the AT-3 BC cell line [161]. Moreover, CSC subpopulations in NOD/SCID mice were more likely to be tumorigenic than non-CSCs [162]. This finding suggests that CSCs have a potential NK cell-resistant phenotype to evade NK cell-mediated cell death. The mechanism by which CSCs evade NK cell surveillance involves the upregulation of MHC-I molecules by CSCs to acquire NK cell resistance [163]. Accumulated data suggest that HLA-G and HLA-E send inhibitory signals to NK cells after binding ligands that also contribute to CSC immune evasion [164]. CSCs resist NK cell-mediated cell death by disrupting the balance between NK-activating ligands and inhibitory receptors in BC [165]. This crosstalk between CSCs and NK cells causes changes in ligands and markers on the surface of NK cells, resulting in a selective loss or decrease in NK cell cytotoxicity and a functional state of "division incompetence" [166].

### Hypoxic microenvironment

Evading immune destruction is a fundamental hallmark of cancer cells [167]. Cancer cells produce a heterogeneous TME through multiple metabolic pathways to evade the immune surveillance of NK cells [8]. The prevalent hypoxia in the TME is thought to be a common factor in tumour immune evasion [168]. Under hypoxia, NK cells undergo functional reprogramming, which profoundly affects the nature of NK cell infiltration and the immune-mediated response within the tumour tissue [169]. Mitochondrial disruption, downregulation of NK cell-activating receptors, reduction of perforin, and degradation of NK cell-derived granzyme B are central mechanisms contributing to impaired NK cell effector function under hypoxia [170–172].

Cells rely on the activation of hypoxia induced transcription factors (HIFs) to adapt to the hypoxic environment, especially in response to normoxia-hypoxia, and as a result, new metabolic features and immunosuppressive microenvironments are generated [5, 173]. Loss of HIF-1α promotes the expression of NK activation markers and effector molecules and enhances the response of NK cells to IL-18. HIF-1α inhibitors enhance antitumour activity in NK cells [174]. Moreover, another study highlighted that conditional deletion of HIF-1α in NK cells reduces tumour progression by inducing non-productive angiogenesis in tumours [175]. Overall, these studies reveal that the regulation of NK cell function by hypoxic signalling in the TME is undoubtedly an important factor influencing the therapeutic response of NK cells.

### Metabolic microenvironment and acidic microenvironment: focusing on glycolysis and lactate metabolism

Glucose is a key energy source for cytotoxic NK cells, which require glucose-driven glycolysis and oxidative phosphorylation (OXPHOS) to meet energy requirements [176]. Growing evidence shows that the high glycolytic capacity and glycolytic reserve of NK cells are critical to supporting the antitumor activity of NK cells [177–179]. Deregulating cellular energetics is another important hallmark of cancer cells [167]. Under aerobic conditions, cancer cells use glucose for glycolysis, a process known as aerobic glycolysis or the Warburg effect [180]. Under metabolic stress, cancer cells inevitably compete with NK cells for limited glucose, and tumour-driven glucose restriction may reduce glycolysis in NK cells, thereby impairing their antitumour function. Metabolic competition is thus considered an important driver of tumour progression [181]. Various strategies have been used to overcome the "Warburg effect" in cancer cells to enhance the antitumour effect of NK cells [182].

The regionalized distribution of the vascular system within solid tumours allows for metabolic heterogeneity in cancer cells. New evidence suggests that lactate produced by glucose consumption in hypoxic tumour regions via anaerobic glycolysis can be used as an energy source for the tricarboxylic acid (TCA) cycle in adjacent cancer cells [183]. Lactate is no longer simply considered a metabolic waste; its newly found role in cancer cells is reshaping the field of energy metabolism [184]. Although cancer cells can respond to extracellular acidosis and redox reactions by regulating the expression of monocarboxylate transporters (MCT) to adapt to survival and proliferation, exposure to a high lactate environment damages the effector function of NK cells. Preliminary evidence suggests that lactate impairs NK cell function and survival, thereby causing immune evasion and tumour progression [185]. Similarly, the accumulation of lactate in the TME leads to mitochondrial dysfunction and apoptosis of liver-resident NK cells, and the depletion of liver-resident NK cells leads to CRC liver metastases [186]. Tumour-derived lactate can inhibit the cytolytic function of NK cells through direct or indirect pathways involving decreased expression of NK cell perforin, granzyme, and NKp46 and increased abundance of MDSCs [187]. Lactate accumulated in the TME has been considered a potential antitumor target, but this discovery faces significant challenges in translating into clinical therapy [188]. Comprehensive analysis of lactate metabolic signalling and the interaction of lactate with other components in TME, especially NK cells, may be a promising strategy to overcome the limitations of immunotherapy, and more studies are needed.

### Innervated niche

The "innervated niche" is an emerging concept that aims to describe a specific biological niche "governed by nerves" that is established by the crosstalk between nerves and tumours. There are four main types of cancer-nerve interaction: electrochemical interactions, paracrine interactions, systemic neural-cancer interactions, and cancer therapy-nervous system interactions [189]. Neurological modulation of the immune system and cancer immunotherapy effects on the nervous system represent different mechanisms of neural-cancer crosstalk.

**Fig. 4:**
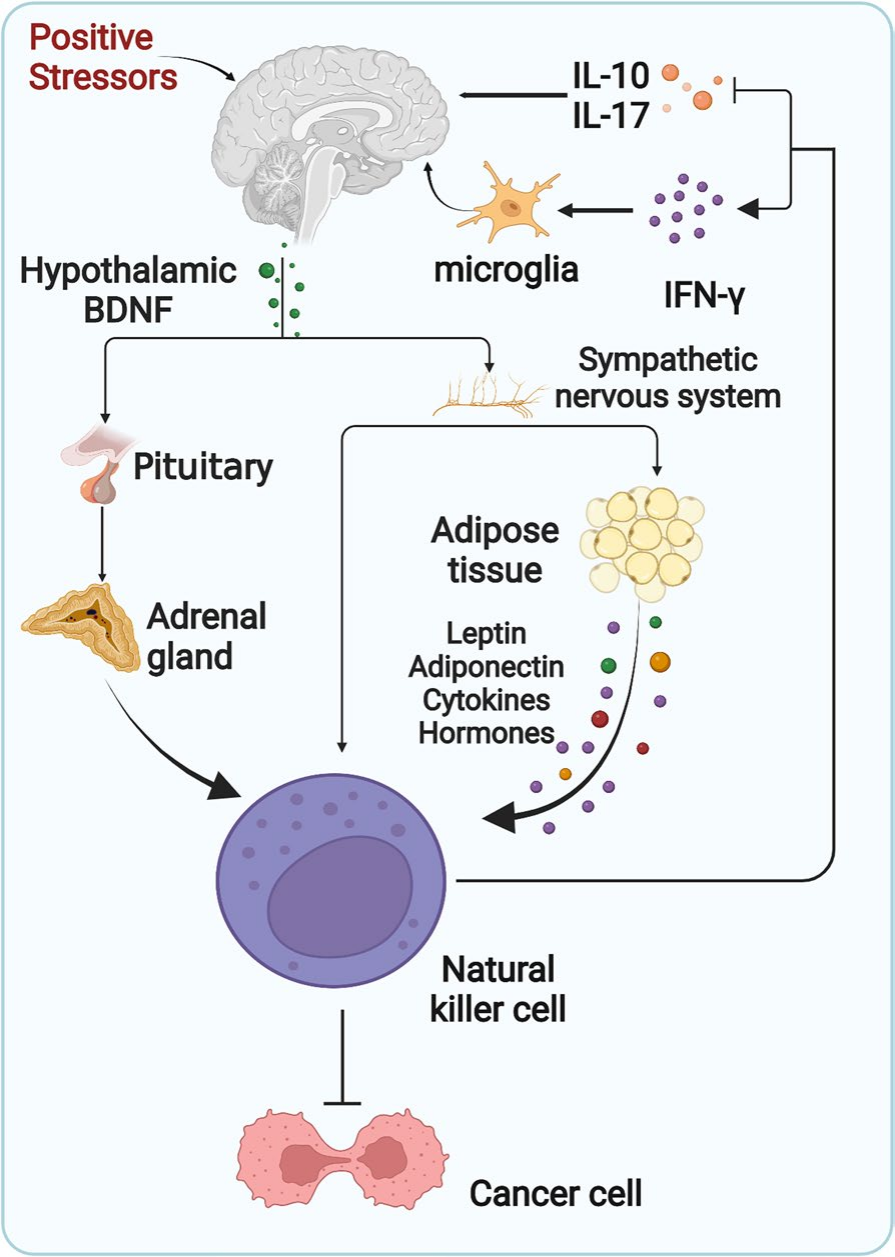
Mechanisms of innervated niche crosstalk with NK cells. Positive stressors enhance the antitumor effects of NK cells via the hypothalamic–pituitary–adrenal axis or the sympathetic nervous system. Likewise, NK cells have positive effects on the nervous system. IFN-γ, Interferon-γ. IL-10/17, Interleukin-10/17. BDNF, Brain-derived neurotrophic factor. *Adapted from “Hypothalamic-Pituitary-Organ Axis with Cellular Effect (Layout)”, by BioRender.com (2022). Retrieved from *https://app.biorender.com/biorender-templates

Environmental enrichment (EE) is an established stress model for studying neurogenesis and brain plasticity. Increasing evidence shows that EE can affect the phenotype and function of NK cells. For example, EE positively regulates the maturation, proliferation, cytotoxicity, and tumour infiltration capacity of NK cells, and mice exposed to EE exhibited an antitumour phenotype [190]. EE exposure selectively upregulates hypothalamic brain-derived neurotrophic factor (BDNF) and other brain mediators, induces sympathetic nervous system (SNS) and hypothalamic–pituitary–adrenal axis (HPA) activation, and directly targets NK cells. Moreover, EE acts on adipose tissue and other endocrine organs, which regulate NK cells by secreting leptin, adiponectin, cytokines, and hormones [191]. These mechanisms contribute to the role of NK cells as key mediators in EE-tumour crosstalk and emphasise the significance of a positive stress response and positive emotions for improving immune function and possible antitumour effects (Fig. 4). In contrast, negative stress responses (forced swimming, abdominal surgery) induce inhibition of NK cell activity, which is a major mediator of tumour progression in stress patterns [192]. In population studies, psychological stressors (such as divorce) have also been associated with reduced NK cell activity and increased risk of cancer [193].

The effects of NK cells on the nervous system are also worth exploring. Under the recruitment of chemokines (CX3CL1, CCL2, and CXCL10) secreted by central nervous system (CNS) resident cells (microglia, astrocytes, and neurons), circulating NK cells enter the CNS through the blood–brain barrier (BBB) and the choroid plexus, settle in the CNS parenchyma, and become a small proportion of the CNS immune cells [194]. There is growing evidence that NK cells ameliorate a variety of neurodegenerative diseases, including Alzheimer’s, Parkinson’s, and multiple sclerosis. Degradation and internalization of neurotoxic aggregates such as alpha-synuclein (α-syn) protein by NK cells [195], activation of microglia with neuroprotective effects by IFN-γ secreted by NK cells [196], suppression of myelin-reactive Th17 cells [197], and inhibition of inflammatory cells by IL-10 are among the mechanisms involved. NK cells have demonstrated neurotoxic effects via mechanisms such as IFN-γ-mediated cytotoxic T cells, DC recruitment and priming, inflammatory cells promotion via cytokines such as granulocyte–macrophage colony stimulating factor (GM-CSF), and direct release of perforin and granzyme [198]. These findings highlight the elusive dual role of NK cells in CNS homeostasis. It is unclear whether NK cells contribute to the CNS disease pathogenesis or outcome, and this dual role is more likely to be related to individual disease stages and CNS-resident NK cell phenotypes. Overall, the immunomodulatory role of NK cells in mediating cancer-neural crosstalk is critical for cancer progression, and more research is needed to further characterise neural-NK cell-cancer regulatory networks.

### Mechanical microenvironment

The TME, as a dynamic and heterogeneous integrated environment, consists mainly of biochemical and mechanical microenvironments. In recent years, researchers have begun to focus on the regulation of the mechanical microenvironment in the TME. Recent studies have shown that the biological behaviour of NK cells in the TME is influenced by mechanical signals, including adhesion, migration, tissue infiltration, and cytotoxic functions [199].

The infiltration of NK cells is accompanied by dramatic changes in the mechanical properties of the stroma and target cells, including cancer cell solid stress, ECM stiffness, topography, and fluid stress [200]. Altered mechanical properties affect the cytotoxic effects of NK cells. It was observed that the softness or hardness of the substrate did not affect the extent of NK cell degranulation; however, exposure to hard substrates enhanced the cytotoxic effect of NK cells, suggesting that mechanosensitivity mediates the cytotoxic effect of NK cells [201]. The decreased rigidity of invasive cells leads to the inability of NK cells to fully respond, which represents a novel mechanism by which cancer cells evade NK cell surveillance [201].

Dynamic actin-mediated mechanical signals are essential to maintain the effector function of NK cells. Evidence suggests that conformational changes in SH2-domain-containing protein tyrosine phosphatase-1 (SHP-1) mediated by actomyosin retrograde flow (ARF) regulate NK cell cytotoxicity. NK cell-activating receptors cause rapid actin flow, which prevents SHP-1 from binding to the actin network, and SHP-1 maintains an inactive conformation and initiates cancer cell death. Conversely, inhibitory receptors slow actin flow, and SHP-1/actin binding activates the SHP-1 conformation, thereby inhibiting NK cell activation [202]. Similarly, actin associated with cytoskeletal conformational rearrangements is actively involved in the formation of lytic IS in NK cells [203]. In conclusion, an understanding of NK cell mechanical signal regulation is necessary for the construction of in vitro mechanical microenvironment models, which are essential for evaluating future NK cell therapies.

### Microbial microenvironment

The microbial microenvironment, which has been an underestimated part of the TME for a long time, has now been shown to form a multidimensional tumour-immune-microbe biological network and to be a potential area for improving tumour immunotherapy [204]. Cumulative data suggest that multiple probiotics in the gut enhance NK cell activity and function by promoting the expression of inflammatory cytokines by NK cells [205]. The microbe-mediated immunomodulatory effects of NK cells are also being investigated in the TME.

Microbial regulation of NK cells can alter tumour progression. In a carcinogen-induced in situ mouse model of CRC, colonisation of *Helicobacter hepaticus* (Hep) resulted in increased infiltration of NK cells and reduced tumour load [206]. Interestingly, Lam et al. found that microbiota could switch the tumorigenic preferences of the microenvironment. A favourable microbiota (intact microbiota, high-fibre diet, responder patients) triggers the production of IFN-I by intratumoral monocytes and regulates macrophage polarisation as well as DC-NK cell crosstalk to form an antitumour microenvironment. In contrast, when the microbiota are adversely disrupted, the monocyte-IFN-I-NK cell-DC cascade is disrupted, and macrophages display the protumour phenotype, creating a protumour microenvironment [207].

The regulation of NK cells by microbiota can affect tumour metastasis. It has been observed that the growth of melanoma cells in bone triggers the proliferation of intestinal NK and Th1 cells and their homing to tumour-bearing bone to inhibit melanoma bone metastasis. This trigger is microbially dependent, and it is weakened by microbiome depletion, thus increasing the progression of bone metastasis [208]. Similarly, Yin et al. found that *Fusobacterium nucleatum* promotes CRC liver metastasis by increasing the accumulation of MDSCs and Tregs in the liver of a CRC mouse model and reducing the infiltration of NK cells to inhibit the immune niche of the liver [209]. Consistently, the results of Gur et al. showed that *Fusobacterium nucleatum* adhering to various cancer cells inhibited NK cell cytotoxicity by binding to the NK cell inhibitory receptor TIGIT [210].

Although the beneficial effect of microbiota on NK cell activity still needs further exploration, it represents a new immunomodulatory mechanism.

## NK cells as potential participants in tumour metastasis: interaction of NK cells with the metastatic niche

Metastasis is a multistage process that comprises three phases: dissemination, dormancy, and colonization. Metastasis is initiated and maintained by a subpopulation of cancer cells with a stem cell-like phenotype and immune evasion properties, called metastasis-initiating cells (MICs). During propagation and dormancy, MICs are in dynamic balance with host immunity. Once immune surveillance fails, metastasis and organ colonisation will occur [211]. The primary tumour and distant premetastatic sites form a metastasis-friendly microenvironment, termed the premetastatic niche (PMN), which has been recognised as an important factor in immune surveillance failure [212]. NK cells have long been characterised as powerful mediators of cell death that directly eliminate cancer cells and inhibit tumour metastasis. However, recent studies have shown that NK cells can exhibit a prometastatic state [213]. Understanding how PMN promote the prometastatic state of NK cells will aid in the refinement and optimization of NK cell antitumour strategies.

Hematogenous metastasis is the main route of metastasis for most tumours. Primary cancer cells invade and intravasate into new capillaries, shedding circulating tumour cells (CTCs), which travel from the circulation into a new host parenchyma to complete the metastasis of the primary tumour to regional and distal sites. In the circulation, cancer cells must overcome shear stress, oxidative stress, and immune cell attack [211]. PMN remodel the vascular state to aid CTC colonisation during this process. A single CTC in the intravasation cycle is often cleared by NK cells, while CTC clusters are protected from NK cell attack by platelet coats and platelet/neutrophil-associated clusters [214, 215]. This observation underscores the unique role of platelets in protecting cancer cells from NK cell attack, and a significant decrease in NK cell-mediated tumour cell survival in Galphaq (a G protein essential for platelet activation) deficient mice has also been observed [216]. Lo et al. reported the mechanism by which CTC cluster resistance to NK cell immunosurveillance in a follow-up study. The metastatic advantage of CTC clusters is associated with their alteration of cell adhesion and epithelial-mesenchymal properties [217].

Metastatic dormancy is the balance between cancer cell cycle arrest, attempted proliferation, and colonization. Dormant cancer cells adapt to niches and establish complex interactions with immune cells. Massagué summarised that dormant MIC metastasis necessitates not only overcoming systemic immunity of NK cells, resident immunity, and reactive stroma, but also adapting to the phenotype required for dormancy, which includes immune suppression adaptations such as perivascular niches, immune evasive dormancy, TGF-β inhibition, metabolic adaptation, and reactive stroma blockers [211]. It has been observed that MICs can initiate a dormancy programme themselves by autocrine signalling of DKK1 to inhibit WNT signalling and bring MICs into a quiescent state. Quiescent MICs downregulate the expression of NK cell-activating ligands to evade NK cell-mediated clearance [218]. In a latent MICs model, re-entry of latent MIC cells into a proliferative state triggered the expression of NKG2D, and latent MIC cells were cleared by NK cells. Similarly, in a NK cell depletion model, depletion of NK cells to allow progressive growth of metastatic lesions was observed [219]. Interestingly, NK cells can "chew off" a portion of the cell membrane carrying a surface molecule (such as PD-1) of the donor cell membrane from an antigen-presenting cell through trogocytosis, thus entering a dormant state and relieving the anticancer activity [220]. In addition, Correia et al. found in dormant disseminated tumour cells (DTCs) that decreased numbers of NK cells in the liver caused a large proportion of cancer cells to metastasize to the liver and that amplification of NK cells with IL-15 caused the cancer cells to enter a dormant state where metastasis was prevented [221].

Although the interaction between NK cells and the metastatic niche complicates the exploration of how cancer cells in dormancy evade NK cell-mediated cell death, the role of NK cells in maintaining tumour dormancy is gaining attention and represents a promising prospect for antimetastatic drug development.

## Current therapeutic strategies for overcoming the TME

### CAR-NK

The concept of chimeric antigen receptors (CAR) was pioneered by Gross and his colleagues in 1989. After decades of development, CAR-T cell therapy has made many breakthroughs, and the design of CAR has been gradually optimized. Compared with T cells, NK cells are more advantageous in the development of a CAR engineering platform due to their broad-spectrum antitumour activity, relative safety, broad accessibility, and allogeneic use [7]. Based on the unique advantages of CAR-NK technology, many preclinical and clinical trials have been conducted to evaluate the therapeutic value, safety, and manufacturing process of CAR-NK technology and to actively summarise the shortcomings and challenges of CAR-NK cell therapy.

Various strategies have been developed to enable CAR-NK cell therapy to deregulate the suppressive TME. In recent years, combinations of cytokines have been widely used to stimulate NK cell proliferation and improve persistence, such as the simultaneous expression of IL-15 and other molecules on CAR-NK cells [222]. CAR design targeting TME products (e.g., lactate, adenosine, ROS, etc.) and the hypoxic microenvironment represents an innovative CAR construction concept that may be a new weapon to enhance CAR-NK immunotherapy in solid tumours. For example, a genetic modification strategy that involves terminating the release of immunosuppressive metabolites (e.g., ROS) in solid tumours was used to enhance the resistance and efficacy of CAR-NK cells against solid tumours [223]. A CAR incorporating the oxygen-sensitive structural domain of HIF-1α (HIF-CAR) can generate CAR constructs responsive to hypoxic environments [224]. Depolarized or repolarized immunosuppressive cells such as CAFs, TAMs, and MDSCs have also shown convincing efficacy in preclinical models of CAR-T cells [225–227]. It is also an attractive option to enhance CAR-NK immunotherapy in the future.

### NK cell engagers

Unlike CAR-NK cell therapy, which expresses CAR on NK cells to mediate the targeted killing of tumour cells, NK cell engagers (NKCEs) consist of a single-chain fragment variable (scFv) of antibodies against NK cell activation receptors (e.g., CD16, NKp30, NKp46, and NKG2D) and one (Bispecific killer engagers, BiKEs) scFv of antibodies against different tumour antigens [228]. This "two-pronged" strategy allows the effective binding of NK cells and cancer cells together, forming IS and conferring specific killing activity to NK cells [229]. The introduction of more scFvs or the insertion of IL-15 as a linker, assembled into tri-or even tetra-specific killer cell engagers (TriKEs and TetraKEs), further enhanced NK cell proliferation and survival [230]. In preclinical studies, NKCEs have been used effectively in CD33^+^ acute myeloid leukaemia [231] and myelodysplastic syndromes [232], epidermal growth factor receptor (EGFR) in multiple cancers [233], B cell maturation antigen (BCMA) in multiple myeloma [234], etc.

Although most NKCEs are currently in the preclinical stage and their safety and efficacy need further evaluation, it is undeniable that they are emerging as highly promising tumour therapeutic strategies and have the potential to overcome the suppressive TME due to their target specificity [235]. It has been observed that 161,533 TriKE exhibits excellent antitumour activity and promotes NK cell persistence in vivo [236]. Clinical trials applying NKCEs to a variety of hematologic malignancies have demonstrated potential antitumour effects (NCT01221571, NCT01221571) or are recruiting (NCT03214666, NCT04101331).

### Immune checkpoint inhibitors

Antitumour immunity of NK cells is a "gambling" process of dynamic balance between NK cells and cancer cells, in which the complex interactions of multiple activating or inhibiting receptors or ligands form the basis of NK cell recognition and activation [237]. Cancer cells exploit NK cells' inhibitory receptors for immune escape, and immune checkpoint inhibitors (ICIs) reactivate NK cells by relieving this inhibition.

A variety of NK cell checkpoint receptors are becoming popular targets for tumour immunotherapy [238] and have demonstrated success in terms of safety, tolerability, and survival [239, 240]. However, the efficacy of immune checkpoints in the TME in patients with solid tumours needs further evaluation, and single checkpoint receptor blockade may not be sufficient to completely rescue NK cells within the TME, which express multiple immune checkpoint ligands [241]. Multiple combination strategies need to be tried to overcome the TME, such as ICIs combined with surgery, chemotherapy, radiotherapy, targeted therapy, and other therapies, which are gradually becoming a popular research direction.

### Nanoparticles

Accumulated evidence has shown the great potential of nanoparticles in enhancing NK cell-mediated antitumour immunity, making it possible to unlock numerous innovative approaches to inhibit the microenvironment in the TME. For example, nanoscale liposomes and nanoemulsion system could be used to deliver TGF-β inhibitors and modify TME [132, 242]. Nanoparticle strategies have also been developed to improve the homing and infiltration properties of NK cells, such as liposomes loaded with TUSC2 or nanocomposite microspheres encapsulated with IFN-γ to induce a significant increase in NK cell infiltration [243, 244]. The application of an external magnetic field also helps to guide NK cells bound to magnetic nanoparticles into the tumour, and increase NK cell infiltration [245]. There are also cases of applying nanocarriers to silence NK cell inhibitory signals to release NK cell activity and using multi-targeted nano-junction platforms to promote NK cell recruitment and activation [246, 247].

### Small molecules

Small molecules' ability to easily cross cell membranes to access intracellular targets makes them more permeable to the TME, giving them an inherent advantage in overcoming the TME. Zhong et al. reviewed the current application of small molecules in interrupting the hypoxic, acidic, and inflammatory environments in the TME as well as the aberrant ECM network, stating that small molecules can be an attractive strategy for targeting TME to enhance tumour therapy [248].

It has been proven that a variety of small molecules can regulate the antitumour function of NK cells by changing the balance of NK cell activation and inhibition signals or by participating in the expansion, activation, differentiation, and maturation of NK cells [249]. In conclusion, the application of NK cell immunotherapy using small molecules is worthy of anticipation and further research.

## Outlooks and directions

In the previous chapters, we discussed the interaction between NK cells and specialised TME, which formed an intricate crosstalk network. Given that targeting TME-NK cell crosstalk represents a promising direction for tumour therapy, a number of questions have been raised.

**Fig. 5:**
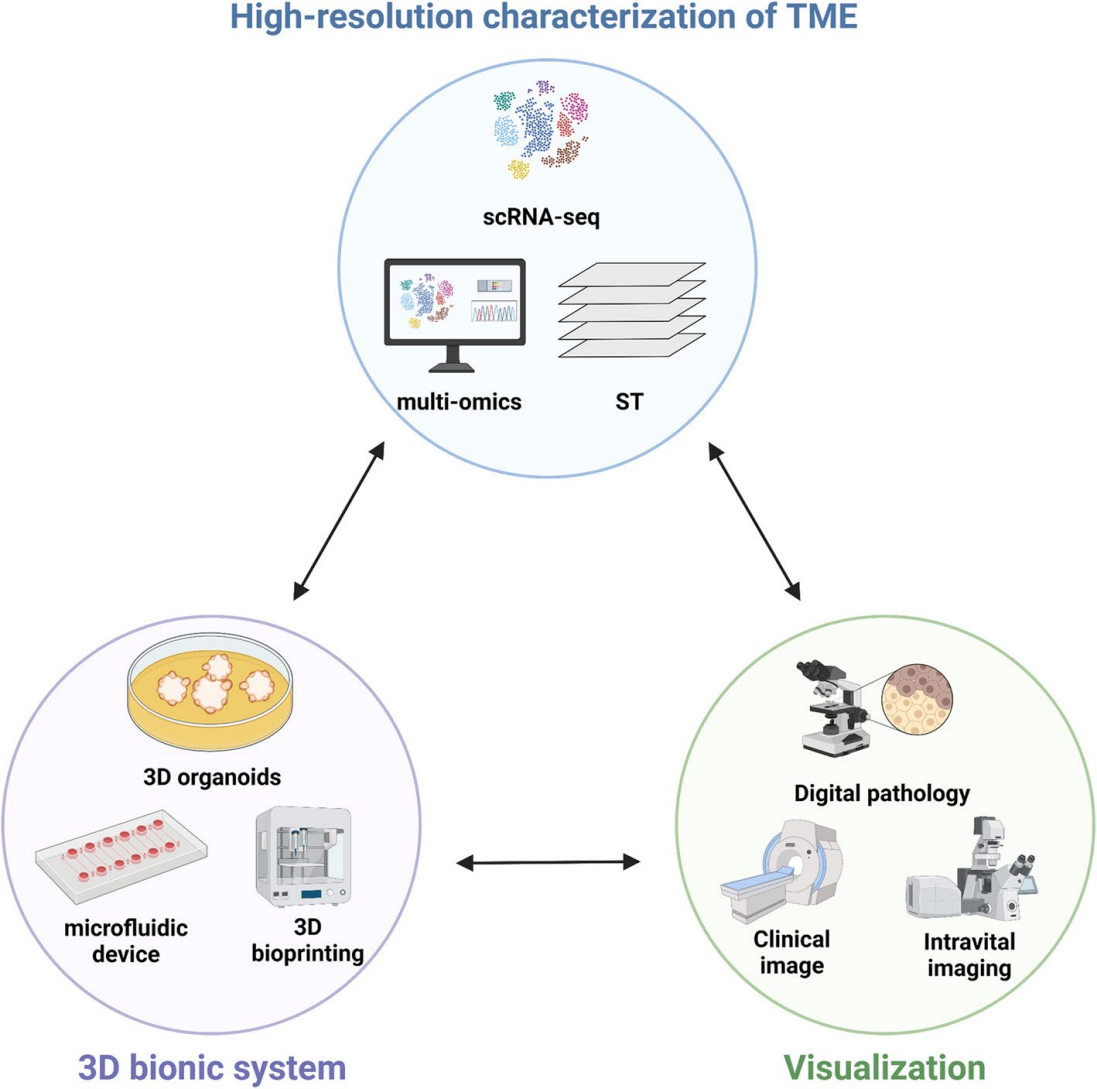
Directions and outlooks for overcoming inhibitory microenvironments. ST, Spatial transcriptomics. *Adapted from “Components of Tissue Engineering”, by BioRender.com (2022). Retrieved from *https://app.biorender.com/biorender-templates

First, higher-resolution methods are needed to characterise the heterogeneous subsets, spatial distribution, and functional status of NK cells in TME (Fig. 5). Single-cell RNA sequencing (scRNA-seq) makes it possible to characterise specific cell populations and cell subset states, and is a powerful tool for dissecting heterogeneous TME [250, 251]. An unanswered question, however, is why scRNA-seq does not fully map the spatial location of cells in involuted TME. The technology that combines scRNA-seq with spatial transcriptomics (ST) emerges as a solution to this problem [252]. ST can realise the spatial visualisation of transcriptome, and it is worth looking forward to applying it to the TME cell-to-cell crosstalk. To surmount the limitations of single omics analysis, scRNA-seq methods combined with multi-omics (transcriptomics, proteomics, epigenomics, and metabolomics) methods were developed [253]. Integrative analysis of single-cell multi-omics data reveals cellular heterogeneity in the TME across multiple molecular dimensions, providing more novel insights into the identification of specific cell subsets, molecular features, and underlying mechanisms that mediate changes in cellular functional state.

Second, in vitro research models need to be established to simulate and decipher specific interactions between specializsed TME and NK cells (Fig. 5). Microfluidic in vitro models have been shown to be a simple alternative tool for studying cancer-NK cell interactions, with the benefits of real-time monitoring of NK cell infiltration and simulating the direct contact between NK cells and cancer cells [254, 255]. Three-dimensional (3D) organoids and 3D bioprinting have advantages in displaying multicellular structures and complex positions of cells in the TME and have been developed for 3D reconstruction of complex microenvironments [200, 256, 257]. The above 3D bionic system allows for the in vitro reconstruct for the complex microenvironment, which is a dynamic crosstalk with NK cells.

Last but not least, it is necessary to visualise the functional status of NK cells at different clinical stages and under different experimental characteristics (Fig. 5). In the panoramic analysis of TME and the characterization of cell spatial location, traditional protein techniques, including immunohistochemistry and immunofluorescence, have exposed some limitations. Spatial protein techniques, including multiplex immunohistochemistry (mIHC), multiplex immunofluorescence (mIF), cytometry by time of flight (CyTOF), and multiplex digital spatial profiling (DSP), allow for the simultaneous assessment of multiple protein markers and contribute to the acquisition of a more comprehensive microenvironment map [258]. Live or intravital imaging techniques, such as confocal microscopy, two-photon microscopy, and fluorescence-based biosensors, have shown attractive potential in subcellular dynamic tracking [259, 260], allowing 3D monitoring and visualisation of NK cell interactions in the TME.

## Conclusion

NK cells naturally recognise self and non-self, and kill cancer cells through multiple lytic pathways, representing a powerful tool for cancer immunotherapy. Recognizing that TME is often an important driver of NK cell dysfunction as well as the immune escape of cancer cells, a deep understanding of the logic of TME-NK cell crosstalk is necessary to propose new therapeutic strategies.

Here, we systematically discuss TME-mediated changes in NK cell functions from different classifications of TME based on multiple perspectives. Despite the progress made, our characterization of the functional state of NK cells in TME is poorly understood or even lacking. It is difficult to define whether NK cells are friends or foes. NK cells cooperate with or fight against other participants in the TME, and together they form a complex TME ecosystem. Just as NK cells can shift from a tumour killer to a pro-tumour metastatic state, a more plausible explanation is that NK cells switch roles at different stages of cancer. Despite representing therapeutic potential, lack of evidence from clinical trials remains the greatest challenge in targeting TME-NK cell crosstalk.

## Data Availability

Not applicable.

## Abbreviations

ECM: Extracellular matrix
TME: Tumour microenvironment
CAFs: Cancer-associated fibroblasts
MSCs: Mesenchymal stem cells
CSCs: Cancer stem cells
NK: Natural killer
ADCC: Antibody-dependent cell-mediated cytotoxicity
FasL: Fas ligand
TRAIL: TNF-related apoptosis-inducing ligand
KIRs: Killer immunoglobulin-like receptors
LIRs/ILTs: Leukocyte Ig-like receptors
MHC: Major histocompatibility complex
NCR: Natural cytotoxic receptors
DNAM-1: DNAX accessory molecule 1
NKG2D: Natural killer group 2 member D
GJ: Gap junctions
Cx: Connexins
ATP: Adenosine triphosphate
cAMP: Cyclic adenosine 3',5'-monophosphate
IP3: Inositol triphosphate
IS: Immune synapses
dNK: Decidual NK
TDEs: Tumour-derived exosomes
NK-Exos: NK cell-derived exosomes
IL: Interleukin
BC: Breast cancer
cDC1: Conventional type 1 dendritic cells
IFN-γ: Interferon-γ
Th1: T helper cell type 1
HNC: Head and neck cancer
MICA/MICB: MHC class I chain-related protein A/B
Tregs: Regulatory T cells
NSCLC: Non-small-cell lung cancer
A2AR: A2A adenosine receptor
CTLA-4: Cytotoxic T-lymphocyte antigen 4
PD-1: Programmed death-1
CCR4: C-C chemokine receptor type 4
STAT3: Signal transducer and activator of transcription 3
DCs: Dendritic cells
FLT3L: FMS-related tyrosine kinase 3 ligand
cDC: Conventional DC
CXCL9/10: Chemokine (C-X-C motif) ligand 9/10
TNF-α: Tumour necrosis factor α
PGE2: Prostaglandin E2
EVs: Extracellular vesicles
ROS: Reactive oxygen species
NETs: Neutrophil extracellular traps
TAMs: Tumour-associated macrophages
pTA-NK: Peripheral blood tumour associated circulating NK
pCAFs: Cancer-promoting CAFs
rCAFs: Cancer-restraining CAFs
IDO: Indoleamine 2,3-dioxygenase
PVR/CD155: Poliovirus receptor
HLA: Human leukocyte antigen
HIFs: Hypoxia induced transcription factors
OXPHOS: Oxidative phosphorylation
TCA: Tricarboxylic acid
MCT: Monocarboxylate transporters
MDSCs: Myeloid-derived suppressor cells
EE: Environmental enrichment
BDNF: Brain-derived neurotrophic factor
TGF: Transforming growth factor
SNS: Sympathetic nervous system
HPA: Hypothalamic-pituitary-adrenal axis
CNS: Central nervous system
α-syn: Alpha-synuclein
GM-CSF: Granulocyte-macrophage colony stimulating factor
SHP-1: SH2-domain-containing protein tyrosine phosphatase-1
ARF: Actomyosin retrograde flow
CRC: Colorectal cancer
Hep: Helicobacter hepaticus
MICs: Metastasis initiating cells
PMN: Pre-metastatic niche
CTC: Circulating tumour cells
DTCs: Disseminated tumour cells
CAR: Chimeric antigen receptors
NKCE: NK cell engagers
scFv: Single-chain fragment variable
BiKEs/TriKEs/TetraKEs: Bispecific/trispecific/tetraspecific killer engagers
EGFR: Epidermal growth factor receptor
BCMA: B cell maturation antigen
ICIs: Immune checkpoint inhibitors
scRNA-seq: Single-cell RNA sequencing
ST: Spatial transcriptomics
3D: Three-dimensional
mIHC: Multiplex immunohistochemistry
mIF: Multiplex immunofluorescence
CyTOF: Cytometry by time of flight
DSP: Multiplex digital spatial profiling
